# Lipopeptide biosurfactant viscosin enhances dispersal of *Pseudomonas fluorescens* SBW25 biofilms

**DOI:** 10.1099/mic.0.000191

**Published:** 2015-12

**Authors:** Lise Bonnichsen, Nanna Bygvraa Svenningsen, Morten Rybtke, Irene de Bruijn, Jos M. Raaijmakers, Tim Tolker-Nielsen, Ole Nybroe

**Affiliations:** ^1^​ Section for Microbial Ecology and Biotechnology, Department of Plant and Environmental Sciences, Faculty of Science, University of Copenhagen, Copenhagen, Denmark; ^2^​ Costerton Biofilm Center, Department of Immunology and Microbiology, Faculty of Health and Medical Sciences, University of Copenhagen, Copenhagen, Denmark; ^3^​ Microbial Ecology Department, Netherlands Institute of Ecology, Wageningen, The Netherlands

## Abstract

Pseudomonads produce several lipopeptide biosurfactants that have antimicrobial properties but that also facilitate surface motility and influence biofilm formation. Detailed studies addressing the significance of lipopeptides for biofilm formation and architecture are rare. Hence, the present study sets out to determine the specific role of the lipopeptide viscosin in *Pseudomonas fluorescens* SBW25 biofilm formation, architecture and dispersal, and to relate *viscA* gene expression to viscosin production and effect. Initially, we compared biofilm formation of SBW25 and the viscosin-deficient mutant strain SBW25Δ*viscA* in static microtitre assays. These experiments demonstrated that viscosin had little influence on the amount of biofilm formed by SBW25 during the early stages of biofilm development. Later, however, SBW25 formed significantly less biofilm than SBW25Δ*viscA*. The indication that viscosin is involved in biofilm dispersal was confirmed by chemical complementation of the mutant biofilm. Furthermore, a fluorescent bioreporter showed that *viscA* expression was induced in biofilms 4 h prior to dispersal. Subsequent detailed studies of biofilms formed in flow cells for up to 5 days revealed that SBW25 and SBW25Δ*viscA* developed comparable biofilms dominated by well-defined, mushroom-shaped structures. Carbon starvation was required to obtain biofilm dispersal in this system. Dispersal of SBW25 biofilms was significantly greater than of SBW25Δ*viscA* biofilms after 3 h and, importantly, carbon starvation strongly induced *viscA* expression, in particular for cells that were apparently leaving the biofilm. Thus, the present study points to a role for viscosin-facilitated motility in dispersal of SBW25 biofilms.

## Introduction

Bacteria belonging to the genus *Pseudomonas* produce several surface-active molecules, i.e. biosurfactants, that have important functions for the producing cells. The lipopeptides represent a diverse group of powerful biosurfactants that are composed of a lipid tail linked to a short circular or linear peptide (Raaijmakers *et al.*, 2010). This group includes compounds such as amphisin, massetolide, putisolvin and viscosin, characterized in particular for plant- or root-associated *Pseudomonas fluorescens* or *Pseudomonas putida* strains. The lipopeptides initially attracted attention due to their antagonistic effects against fungi and oomycetes (Raaijmakers *et al.*, 2010); however, it is now recognized that they have a broad range of natural roles, including defence against protozoan predators (Mazzola *et al.*, 2009), facilitation of swarming motility and spidery spreading (Alsohim *et al.*, 2014; Andersen *et al.*, 2003; de Bruijn *et al.*, 2007), alteration of soil water characteristics (Fechtner *et al.*, 2011), increased biodegradability of hydrophobic carbon sources (Bak *et al.*, 2015), as well as roles in biofilm formation (de Bruijn *et al.*, 2007, 2008; D'aes *et al.*, 2014; Kruijt *et al.*, 2009; Li *et al.*, 2013; Roongsawang *et al.*, 2003).

Our current information regarding the role of lipopeptide production in biofilm formation does not provide a clear consensus, as *Pseudomonas* lipopeptides appear to play both agonistic and antagonistic roles. Examples of lipopeptides that have been reported to support biofilm formation of the producing strain are massetolide A, sessilin, viscosin and xantholysin (de Bruijn *et al.*, 2007; 2008; D'aes *et al.*, 2014; Li *et al.*, 2013). In contrast, the lipopeptides arthrofactin, orfamide and putisolvin have been reported to decrease biofilm formation (D'aes *et al.*, 2014; Kruijt *et al.*, 2009; Roongsawang *et al.*, 2003).

The above observations were all made using static microtitre plate biofilm formation assays, where biofilm formation is often determined at a single time point only. However, biofilm formation is a dynamic process and involves several steps, such as attachment, microcolony formation, biofilm maturation and dispersal (Pamp & Tolker-Nielsen, 2007).

Detailed studies addressing how lipopeptides affect the temporal dynamics of biofilm formation, and biofilm architecture, are rare. However, a recent study documented that putisolvin plays a role in the structure of mature biofilms and in biofilm dispersal in *P. putida* IsoF (Cárcamo-Oyarce *et al.*, 2015). More detailed information is available for another *Pseudomonas* biosurfactant, i.e. the glycolipid rhamnolipid produced by, for example, *Pseudomonas aeruginosa* PAO1. Rhamnolipids play a role in initial surface colonization, formation of channels between microcolonies and dispersal of *P. aeruginosa* PAO1 biofilms (Boles *et al.*, 2005; Davey *et al.*, 2003; Lequette & Greenberg, 2005; Schooling *et al.*, 2004). Rhamnolipids and lipopeptides produced by *P. fluorescens* and *P. putida* strains have many functional properties in common, such as antimicrobial activity and support of swarming motility (Abalos *et al.*, 2001; Déziel *et al.*, 2003; Haba *et al.*, 2003). We therefore hypothesized that lipopeptides and rhamnolipids have similar roles during biofilm formation.

To test this hypothesis, we focused the present study on the role of lipopeptides in biofilm formation by *P. fluorescens* SBW25 – a model strain often used for studies of bacterial evolution, adaptation and plant colonization (Kassen *et al.*, 2004; Rainey & Rainey, 2003). SBW25 produces the cyclic lipopeptide viscosin, which is composed of nine amino acids coupled to a 3-hydroxydecanoic acid (Raaijmakers *et al.*, 2006). Viscosin is synthesized by a non-ribosomal peptide synthetase encoded by the *viscA*, *viscB* and *viscC* genes (de Bruijn *et al.*, 2007). Previous studies employing microtitre plate assays showed that SBW25 produced more biofilm than the *viscA*, *viscB* or *viscC* mutants impaired in viscosin production (de Bruijn *et al.*, 2007). This functional role of viscosin is opposite to that reported for putisolvin in comparable systems (de Bruijn *et al.*, 2007; Kuiper *et al.*, 2004). Building on these observations, the aims of the present study were to (1) determine the specific role of viscosin in biofilm formation, architecture and dispersal, and (2) relate viscosin effects on the biofilms to timing and localization of *viscA* expression monitored at the single-cell level by the use of a fluorescent bioreporter.

## Methods

### Micro-organisms and growth conditions

Micro-organisms and plasmids used in this study are listed in Table 1. For routine cultivation, *P. fluorescens* SBW25 strains were grown on Luria broth (LB) (content: 5 g yeast extract l^− 1^, 10 g tryptone l^− 1^ and 10 g NaCl l^− 1^) agar plates at room temperature (20–24 °C). Overnight cultures for use in subsequent experiments were cultivated in liquid LB at room temperature on an orbital shaker at 180 r.p.m.

**Table 1.** Strains, plasmids and primers used

Strain, plasmid or primer	Relevant characteristics or sequence	Source or reference
Strains		
*Pseudomonas fluorescens*		
SBW25	WT, produces viscosin.	Rainey & Bailey (1996)
SBW25Δ*viscA*	Impaired in viscosin production: *viscA*::Tn*Mod* Km^r^	de Bruijn *et al.* (2007)
SBW25 Tn*7*::*gfp2*	Produces viscosin; mini-Tn*7gfp* inserted in the chromosome, GFP^+^, Gm^r^	This study
SBW25 Tn*7*::*gfp2ΔviscA*	Impaired in viscosin production: *viscA*::Tn*Mod*; mini-Tn*7gfp*inserted in the chromosome, GFP^+^, Gm^r^	This study
SBW25 Tn*7*::*gfp2*pSEVA237R_P*viscA*	Produces viscosin; mini-Tn*7gfp* inserted in the chromosome, GFP^+^, Gm^r^; contains the pSEVA237R_Pv*iscA* plasmid with the *viscA* promoter sequence fused to a gene encoding the mCherry fluorescent protein	This study
*Escherichia coli* DH5α	Cloning host	Grant *et al.* (1990)
Plasmids		
pSEVA237R	Broad-host-range plasmid, Km^r^	Silva-Rocha *et al.* (2013)
pSEVA237R_Pv*iscA*	*viscA* promoter sequence fused to a gene encoding the mCherry fluorescent protein, Km^R^	This study
Primers		
P*viscA*1 Forward-*Eco*RI	5′-CGATGAATTCTCTCATAAGCCATCTCATCCTTG-3′	This study
P*viscA*1 Reverse-*Bam*HI	5′-CGATGGATCCGGGGCTGTCTGTCACCCTA-3′	This study

### Construction of the *viscA* bioreporter

A 413 bp region upstream of the *viscA* gene (PFLU4007) was amplified by PCR using the primers P*viscA*1 Forward-*Eco*RI and P*viscA*1 Reverse-*Bam*HI (DNA Technology) (Table 1). The fragment containing the *viscA* promoter region was digested with *Eco*RI and *Bam*HI, and inserted into the self-replicating broad-host-range plasmid pSEVA237R (Silva-Rocha *et al.*, 2013) in front of a gene encoding the mCherry protein. The resulting plasmid pSEVA237R_P*viscA* was introduced into *Escherichia coli* DH5α by standard heat-shock transformation. Plasmids were purified from clones resistant to 50 μg kanamycin ml^− 1^. After verifying the presence and correct orientation of the *viscA* promoter in the plasmid by sequencing of a ∼1 kb fragment containing the promoter region and a fragment of the mCherry gene, pSEVA237R_P*viscA* was introduced into WT *P. fluorescens* SBW25 by electroporation and transformants were selected by 50 μg kanamycin ml^− 1^. Subsequently, *P. fluorescens* (pSEVA237R_P*viscA*) was tagged with GFP by introducing the *gfp* delivery plasmid pBK-miniTn*7*-*gfp*2 and the helper plasmid pUX-BF13 by electroporation and selection by 10 μg gentamicin ml^− 1^ (Bao *et al.*, 1991; Koch *et al.*, 2001). Finally, correct insertion and orientation of the Tn*7* transposon was verified by PCR as described in Koch *et al.* (2001).

### Purification of viscosin and quantification by HPLC

Purification of viscosin was performed as described previously by Bak *et al.* (2015). Briefly, *P. fluorescens* SBW25 was cultivated on King's B agar plates (King *et al.*, 1954) in darkness at 28 °C for 1 day before being transferred to 20 °C and incubated for another 3 days. Colony material was suspended in demineralized water (MilliQ; Millipore) and homogenized by shaking. Cells and supernatant were separated twice by centrifugation at 4700 r.p.m. for 20 min at 4 °C in a Sigma 3-18K centrifuge (Sciquip). The supernatant was acidified to pH 2.0 with 1 M HCl and left overnight on ice for a precipitate to form. The solution including the precipitate was centrifuged for 27 min at 7000 r.p.m. and 4 °C in a Sigma 3-18K centrifuge. The supernatant was discarded and the precipitate was washed four times with MilliQ water at pH 2.0. The precipitate was dissolved in MilliQ water and pH was adjusted to 8.0 with 1 M NaOH to fully dissolve the precipitate. The solution was lyophilized and the purity of the lipopeptide preparations was verified by HPLC. HPLC analysis was carried out using a Waters Alliance series 2695 system and a Waters model 996 photodiode array detector. The procedure was carried out as described previously by Bak *et al.* (2015). The same HPLC protocol was used for quantification of viscosin produced in liquid cultures of *P. fluorescens* SBW25.

### Biofilm formation in microtitre trays

The microtitre tray biofilm formation assay was performed essentially as described by O'Toole & Kolter (1998). Briefly, *P. fluorescens* SBW25 and *P. fluorescens* SBW25Δ*viscA* were cultivated in wells of polystyrene microtitre trays (96F; Techno Plastic Products) at room temperature in King's B medium and AB minimal medium supplemented with 10 mM trisodium citrate dihydrate (Merck). AB minimal medium consists of (NH_4_)_2_SO_4_ (15.1 mM), Na_2_HPO_4_ (33.7 mM), KH_2_PO_4_ (22.0 mM), NaCl (0.051 M), MgCl_2_ (1 mM), CaCl_2_ (0.1 mM) and trace metals (100 μl l^− 1^). The trace metal solution contained CaSO_4_.2H_2_O (200 mg l^− 1^), FeSO_4_.7H_2_O (200 mg l^− 1^), MnSO_4_.H_2_O (20 mg l^− 1^), CuSO_4_.5H_2_O (20 mg l^− 1^), ZnSO_4_.7H_2_O (20 mg l^− 1^), CoSO_4_.7H_2_O (10 mg l^− 1^), NaMoO_4_.H_2_O (10 mg l^− 1^) and H_3_BO_3_ (5 mg l^− 1^). Overnight cultures were used as inoculum, resulting in an initial OD_600_ of 0.01. The viscosin production of SBW25 was routinely confirmed by a drop collapse assay (de Bruijn *et al.*, 2007). Cultures were aliquoted (100 μl) into the wells of microtitre trays incubated at room temperature. At specified time points the medium was removed and each well was subsequently washed with 125 μl MilliQ water. Remaining biofilm was stained for 10 min with 150 μl 0.1 % (w/v) crystal violet solution, obtained by dilution of a 1 % stock (Sigma) in MilliQ water. The liquid phase was removed and each well was washed twice with 175 μl of MilliQ water. The plates were left overnight to dry and the crystal violet-stained biofilms were dissolved in 175 μl of 30 % acetic acid for 10 min. The amount of solubilized dye was determined spectrophotometrically at *A*_590_.

### Complementation of *viscA* mutant biofilm with purified viscosin

The liquid phases of 14 h microtitre tray-grown biofilms of *P. fluorescens* SBW25Δ*viscA* were removed. Subsequently 125 μl 50 μg viscosin ml^− 1^ solution in AB minimal medium was added to each well and incubated at room temperature for 9.5 h before assay. AB minimal medium was used as control treatment. The biofilms were quantified by the crystal violet assay as described above.

### *viscA* expression by cells in microtitre plate biofilms

The expression of *viscA* in *P. fluorescens* SBW25 biofilms was assessed by using the fluorescent bioreporter construction *P. fluorescens* SBW25 Tn*7*::*gfp2*pSEVA237R_P*viscA* (Table 1). Briefly, an overnight culture of the reporter strain was diluted to OD_600_ 0.01 in AB minimal medium with 10 mM citrate. The culture was aliquoted (125 μl) into wells of a black-welled polystyrene microtitre plate (MicroWell 96-well optical-bottom, non-treated; Thermo Scientific Nunc) and biofilm was allowed to develop at room temperature as described previously. At certain time points the planktonic phases of the cultures were removed (100 μl) and transferred into new clean wells of the same plate. Fresh AB medium (100 μl) was added to the wells containing the remaining biofilm. OD_450_ and mCherry fluorescence readouts of planktonic cells and biofilm, respectively, were measured as OD_450_ and mCherry units on a Synergy 4 plate reader (xenon flash lamp, laser intensity 225; excitation 587 nm, emission 615 nm; BioTek Instruments). Expression data were calculated as mCherry units/OD_450_, referred to as relative fluorescence units (RFU), in order to normalize *viscA* expression to biomass.

### Cultivation of biofilms in flow cell systems

Biofilms were cultivated in flow cells, which were assembled and prepared as described by Crusz *et al.* (2012). AB minimal medium supplemented with 1 mM trisodium citrate dihydrate was used as growth medium. Individual flow chambers were inoculated by injection of 300 μl aliquots taken from 1 : 1000 dilutions of overnight cultures. Upon inoculation, the flow cells were left upside down without flow for 1 h to allow bacterial attachment to the glass cover. The flow system was incubated at room temperature and a laminar flow with a mean flow velocity of 0.2 mm s^− 1^ was achieved using a Watson Marlow 205S peristaltic pump (Ismatec). Carbon starvation was induced in 1-day-old biofilms by shifting to AB minimal medium without citrate. The biofilms were analysed just prior to the shift of medium and 3 h after.

### Microscopy and image analysis

Image acquisition was performed using a confocal microscope (LSM 710; Zeiss) equipped with an argon and an NeHe laser and detectors, and filter sets for simultaneous monitoring of GFP (excitation 488 nm, emission 517 nm) and mCherry (excitation 587 nm, emission 610 nm). Images were obtained using a × 63/1.4 objective. Simulated fluorescence projections were generated using the imaris software package (Bitplane). For quantitative analysis, six images were taken at random places in biofilms in each of three individual chambers. The analysis was performed with the comstat image analysis software package (Heydorn *et al.*, 2000). A fixed threshold value and connected volume filtration were used for all image stacks.

### Data analysis

Comparison of two datasets was performed by an unpaired two-tailed *t*-test. All experiments were carried out with at least five replicates and at least two independent experiments were conducted. Data are presented as mean ± sd. *P* < 0.05 was selected as the cut-off for statistical significance.

## Results

### Biofilm formation in microtitre trays

**Fig. 1.**
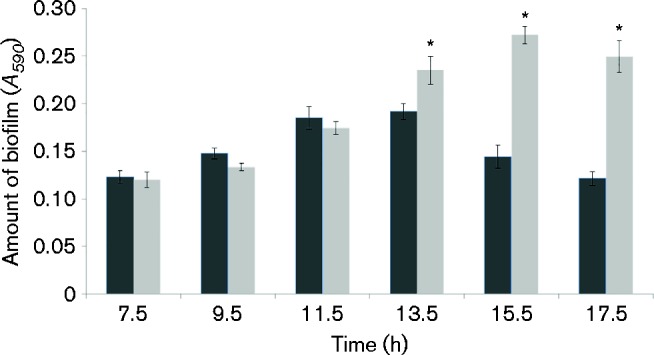
Biofilm formation of *P. fluorescens* SBW25 (dark bars) and *P. fluorescens* SBW25Δ*viscA* (light bars) in AB minimal medium with citrate. The biofilm was quantified from 7.5 to 17.5 h using the crystal violet assay. The *A*_590_ value represents crystal violet-stained biofilm attached to the walls of the microtitre wells and is an indirect measure of the biofilm formed. Data represent mean ± sd for a representative experiment with eight replicates. *Significant difference between strains at each time point (*P* < 0.05).

We initially set out to determine the temporal dynamics of biofilm formation by *P. fluorescens* SBW25 and SBW25Δ*viscA*, a mutant impaired in viscosin production, for cells grown in microtitre plates with AB minimal medium with citrate. The analysis revealed that biofilms of SBW25 and SBW25Δ*viscA* developed comparably until 11.5 h (Fig. 1). Subsequently, a significant difference emerged (*P* < 0.05) as the amount of SBW25 biofilm decreased, whereas the *viscA* mutant continued to develop biofilm throughout the period. Additional assays in King's B medium, to provide a link to previous single-point analyses (de Bruijn *et al.*, 2007), showed that the *viscA* mutant formed more biofilm than the WT strain when incubations were carried out for >24 h (data not shown).

**Fig. 2.**
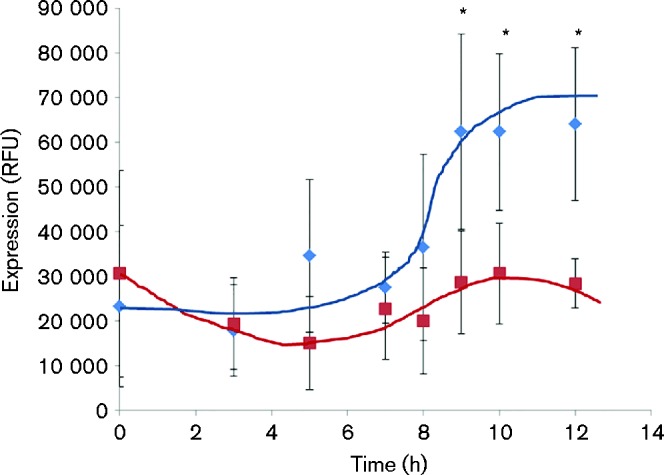
Expression of the *viscA* gene per biomass unit in biofilms (blue diamonds) and planktonic cells (red squares) in the initial state of biofilms grown in microtitre wells. Data represent mean ± sd for a representative experiment with five replicates. *Significant difference between biofilm and planktonic cells at each time point (*P* < 0.05) as well as significant difference from biofilm values at 7 h (*P* < 0.05).

The timing of *viscA* gene expression during biofilm formation by SBW25 was analysed in the initial state of the biofilm formation up until dispersal (0–12 h) by the use of the *viscA* bioreporter strain (Fig. 2). The data for relative *viscA* expression (mCherry fluorescence normalized by OD_450_ as a proxy for biomass) showed that *viscA* was significantly induced in biofilms after 9 h (*P* < 0.05) when values were compared with the 7 h value. Furthermore, *viscA* expression was significantly higher (*P* < 0.05) for biofilms than for planktonic cells after 9 h, i.e. ∼4 h before differences in the amount of biofilm could be observed for SBW25 versus SBW25Δ*viscA*. For the planktonic cells, *viscA* gene expression was relatively stable during the 12 h experiment (Fig. 2).

**Fig. 3.**
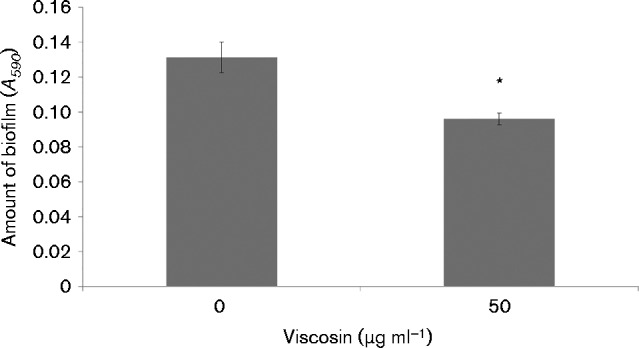
Mature biofilms of *P. fluorescens* SBW25Δ*viscA* complemented with purified viscosin at concentrations of 0 and 50 μg ml^− 1^. Biofilms were quantified after 9.5 h of incubation with and without viscosin by the crystal violet assay. Data represent mean ± sd for a representative dataset including eight replicates. *Significant difference (*P* < 0.05) compared with the control (0 μg ml^− 1^).

To verify that the observed differences between SBW25 and SBW25Δ*viscA* biofilms were associated with viscosin production, mature biofilms of SBW25Δ*viscA* were complemented with purified viscosin at a concentration of 50 μg ml^− 1^, which is comparable to that measured for SBW25 overnight cultures (data not shown). A 9.5 h complementation with viscosin resulted in a significant decrease in the amount of biofilm as compared with the control treatment (*P* < 0.05) (Fig. 3). This suggests that viscosin is, at least in part, responsible for the difference in SBW25 and SBW25Δ*viscA* biofilms.

In conclusion, the experiments based on the microtitre plate assay demonstrated that viscosin has little influence on the amount of biofilm formed by SBW25 during the build-up of biomass, which is in agreement with a low expression of the *viscA* gene. Subsequently, a higher expression of *viscA* was recorded and correlated with an effect of viscosin on biofilm dispersal.

### Biofilm formation in flow cell systems

To analyse the possible involvement of viscosin in dispersal in more detail and to further address whether viscosin has any influence on SBW25 biofilm structure prior to dispersal, we next examined biofilms formed in flow cell systems.

**Fig. 4.**
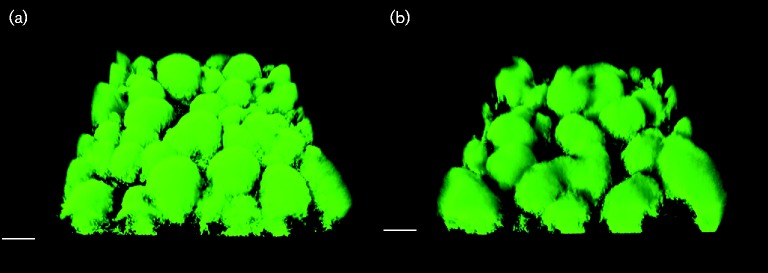
CLSM micrographs of biofilms formed by *P. fluorescens* (a) SBW25 Tn*7*::*gfp2* and (b) SBW25 Tn*7*::*gfp2*Δ*viscA* grown in AB minimal medium with citrate in flow cells for 2 days. Bars, 20 μm.

Rhamnolipid has been shown to play a role in the architecture of *P. aeruginosa* PAO1 biofilms, and we speculated that viscosin could play a comparable role during SBW25 biofilm development. To investigate this, we *gfp*-tagged *P. fluorescens* SBW25 and SBW25Δ*viscA*, and used CLSM to observe the biofilm structures for SBW25 Tn*7*::*gfp2* and SBW25 Tn*7*::*gfp2*Δ*viscA* daily for up to 5 days during cultivation in hydrodynamic flow cell systems. We did not observe any significant difference in the structure of the biofilms formed by the two strains. Both WT and mutant developed a dense biofilm with well-defined microcolonies, as shown in Fig. 4. comstat analysis showed no significant differences (*P*>0.05) in biomass, thickness, surface area or roughness (data not shown).

**Fig. 5.**
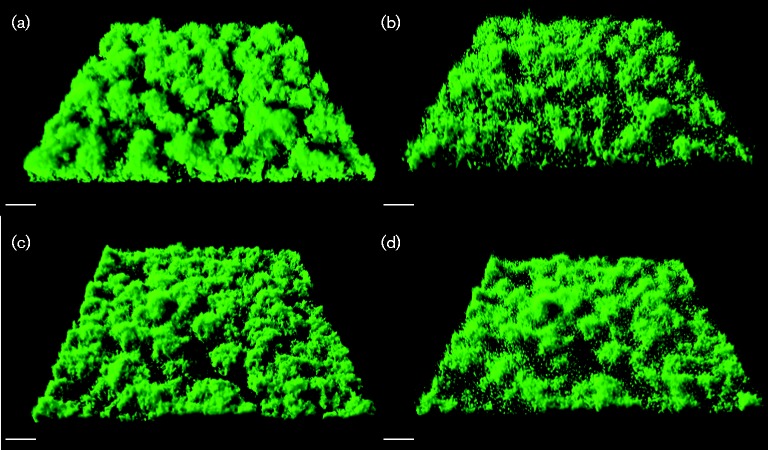
CLSM micrographs of biofilms of *P. fluorescens* (a, b) SBW25 Tn*7*::*gfp*2 and (c, d) SBW25 Tn*7*::*gfp2*Δ*viscA*. The strains were cultivated in flow chamber systems for 1 day with AB minimal medium with citrate, upon which a shift was made to AB minimal medium with no carbon source. Micrographs were acquired immediately before the shift (a, c) and 3 h after the shift (b, d). Bars, 20 μm.

Surprisingly, significant dispersal of SBW25 Tn*7*::*gfp2* was not observed even after incubation for 5 days. We speculated that the flow cell biofilms did not experience sufficient nutrient limitation in contrast to what was experienced by the microtitre well biofilms cultivated under batch conditions. Hence, we imposed carbon starvation on 1-day-old biofilms by shifting to AB minimal medium without citrate. Carbon starvation did indeed induce dispersal of the biofilms formed by the viscosin-producing strain, whereas the same treatment only slightly affected the mutant strain SBW25 Tn*7*::*gfp*2Δ*viscA* (Fig. 5). As the biofilms were visualized by the fluorescence emitted by intrabacterial GFP, differences in the amount of cell biomass were monitored, whereas the experiment could not determine differences in the amount of extracellular matrix.

**Fig. 6.**
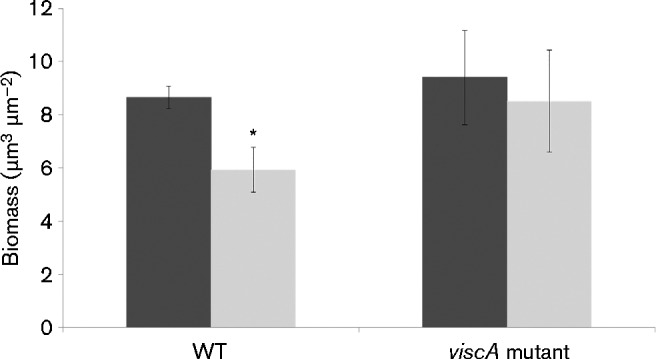
Biofilms of *P. fluorescens* SBW25 (WT) and SBW25Δ*viscA* (*viscA* mutant) were cultivated in flow chamber systems with AB minimal medium with citrate for 1 day, upon which a shift was made to AB minimal medium with no carbon source. CLSM micrographs were acquired immediately before the shift (dark bars) and 3 h after the shift (light bars), and the biomass was quantified using comstat. Data represent mean ± sd of 18 images taken from random places in three independent flow chambers. *Significant difference between pairs of values (*P* < 0.05).

Quantification of the biomass of SBW25 Tn*7*::*gfp2* and SBW25 Tn*7*::*gfp2ΔviscA* before and after 3 h of carbon starvation is presented in Fig. 6. The quantitative analysis confirmed that the SBW25 Tn*7*::*gfp2* biofilm biomass decreased significantly by 32 % (*P* < 0.05), whilst the biomass of the *viscA* mutant biofilms only decreased insignificantly by 10 % (*P*>0.05).

**Fig. 7.**
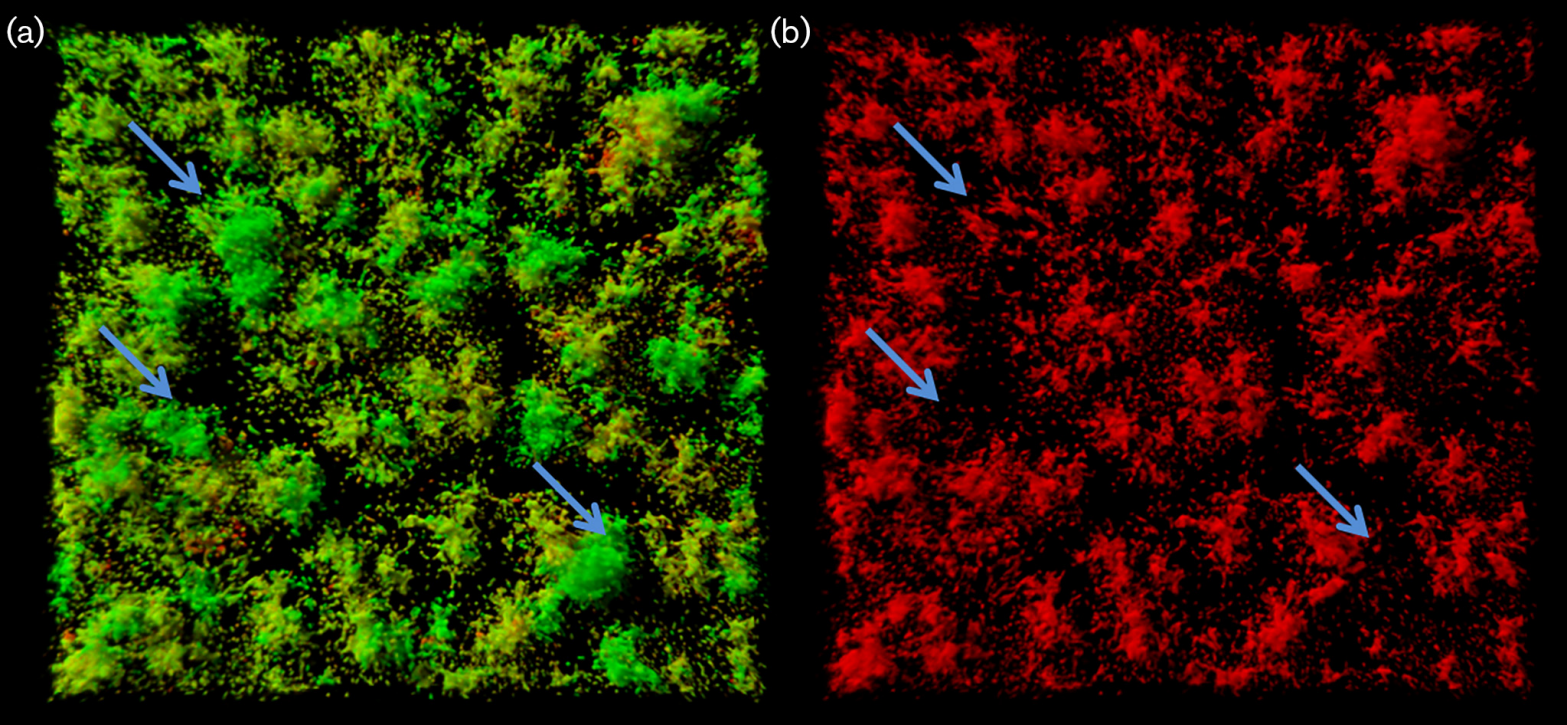
CLSM micrographs of biofilms formed by *P. fluorescens* SBW25 Tn*7*::*gfp*2pSEVA237R_P*viscA.* The images were acquired 3 h after a shift to AB minimal medium with no carbon source. Artificial colours have been assigned in these images to the different CLSM signals; green denotes cells expressing the GFP marker; red denotes cells expressing the *viscA*-mCherry reporter. (a) GFP and mCherry. (b) mCherry only. Arrows indicate selected microcolonies that are only green and not red.

To determine *viscA* expression at the single-cell level in biofilms, we analysed biofilms of the reporter strain SBW25 Tn*7*::*gfp*2pSEVA237R_Pv*iscA* before and after 3 h of carbon starvation. Before the down-shift, *viscA* expression was only observed for a very few cells that appeared to be randomly distributed within the biofilms (data not shown). However, within 3 h of carbon starvation, the mCherry-based *viscA* reporter was turned on. Induced reporter cells occurred throughout the biofilm, and red fluorescent cells were present both at the bottom and in the body of the mushroom structures (seen as red/orange colour in Fig. 7). However, the largest and more developed mushroom structures showed little expression of *viscA* (see arrows in Fig. 7), whereas induced *viscA* reporter cells were primarily associated with smaller structures or were scattered across the substratum.

## Discussion

The present study shows that a viscosin-deficient mutant of *P. fluorescens* SBW25 forms more biofilm than the WT strain over time as determined by microtitre plate assays. Previously, de Bruijn *et al.* (2007) reported the opposite result, but we show here that this apparent discrepancy is due to the temporal dynamics of biofilm formation and dispersal. Hence, SBW25Δ*viscA* consistently forms more biofilm than the WT in long-term incubations.

Several other lipopeptides produced by different *Pseudomonas* species have been characterized by comparison of WT strains and lipopeptide-deficient mutants. Massetolide A, sessilin, viscosin and xantholysin (de Bruijn *et al.*, 2007, 2008; D'aes *et al.*, 2014; Li *et al.*, 2013) have been reported to increase biofilm formation, whilst arthrofactin, orfamide and putisolvin have been reported to decrease biofilm formation (D'aes *et al.*, 2014; Kruijt *et al.*, 2009; Roongsawang *et al.*, 2003). The results presented here suggest that viscosin should be moved to the group of lipopeptides that decrease biofilm formation. However, the current observations strongly indicate that conclusions regarding the effect of biosurfactants on biofilm formation in the standard microtitre assay should be made with caution, as temporal dynamics and assay conditions affect the outcome of assays. Interestingly, viscosin does not appear to play a role in the establishment of viscous mass biofilms at air/liquid interfaces by *P. fluorescens* SBW25 (Koza *et al.*, 2009). Nevertheless, we speculate that more detailed studies may reveal that the roles played by lipopeptides in biofilm formation are not necessarily as conflicting as the current literature indicates.

Our present results indicate that viscosin has an insignificant influence on biofilm structure during the initial phases of biofilm development. No obvious difference was observed when comparing biofilm architecture of the SBW25 WT and *viscA* mutant in the systems used here. This correlates with the low *viscA* gene expression shown by the bioreporter strain. Indeed, fluorescent bioreporter cells were rare and were found scattered in the flow cell biofilms prior to dispersal. These data for early biofilm development are comparable to data obtained for putisolvin, which has a significant effect only on the structure of mature biofilms and where activity of the promoter of the *psoA* biosynthetic gene is found in scattered cells during the early phases of biofilm development in *P. putida* IsoF (Cárcamo-Oyarce *et al.*, 2015).

In contrast, rhamnolipid has been shown to play essential roles in the architecture of developing *P. aeruginosa* PAO1 biofilms (Davey *et al.*, 2003; Pamp & Tolker-Nielsen, 2007). Rhamnolipids play a role during early micro-colony formation, as well as in migration-dependent development of biofilm structures in the later phase of biofilm development (Pamp & Tolker-Nielsen, 2007). Consequently, rhamnolipids and lipopeptide biosurfactants may appear to differ regarding their function during early biofilm development. Importantly, however, both biosurfactant groups play major roles throughout biofilm dispersal. The dispersal mechanism is an important step as it allows bacteria to escape from unfavourable conditions and spread throughout the environment to colonize new areas (Hall-Stoodley *et al.*, 2004).

Our experiments in both the static microtitre well systems and in the hydrodynamic flow cell system demonstrated rapid dispersal of SBW25 biofilms subsequent to expression of the viscosin biosynthetic gene *viscA*, and also that dispersal depended at least in part on viscosin production. One recent study has elegantly shown that quorum sensing induces putisolvin production in mature *P. putida* IsoF biofilms and that biosurfactant production facilitates escape of motile cells from micro-colonies in the biofilm, leading to rapid dispersal (Cárcamo-Oyarce *et al.*, 2015). Similarly, rhamnolipid biosurfactants have been implicated in motility-dependent biofilm detachment by *P. aeruginosa* PAO1 (Boles *et al.*, 2005; Pamp & Tolker-Nielsen, 2007). We observed that SBW25 cells leaving the biofilm were expressing *viscA*, whilst cells remaining in developed microcolonies lacked expression. This could indicate that viscosin-supported motility plays role in dispersal comparable to that shown for putisolvin and rhamnolipids. The observation that *viscA* induction appeared more common in smaller clusters of cells than in the larger biofilm clusters is somewhat surprising, but may be due to the presence of dormant cells within the larger biofilm clusters whose gene expression is not readily induced, or to cluster size-dependent kinetics of the changes occurring after the shift in medium irrigated to the flow cell. However, it should be noted that the regulatory background differs as viscosin production is not under *N*-acyl homoserine lactone-based quorum-sensing control in SBW25 (de Bruijn *et al.*, 2008).

Surprisingly, neither *viscA* gene expression nor biofilm dispersal was observed during 5 days of SBW25 biofilm development in the hydrodynamic flow cell system, whilst both events occurred readily in the static microtitre tray assay. We speculate that in the microtitre plate wells, the cells will experience limitation of nutrients much earlier than in the hydrodynamic flow cell system, and thereby initiate viscosin production. This was also supported by the observation that the *viscA* gene was strongly induced in the flow-cell-grown biofilms when carbon source limitation was enforced.

Dispersal of WT biofilms was induced by introduction of carbon starvation, whereas biofilms of a viscosin-deficient mutant did not disperse to the same extent. Carbon starvation is known to trigger specific biofilm dispersal programmes in several pseudomonads. For example, the LapD and LapG proteins seem to be involved in modification of both cell surface hydrophobicity and biofilm matrix during carbon starvation in *P. putida* OUS82 and *P. fluorescens* Pf0-1 (Boyd & O'Toole, 2012; Gjermansen *et al.*, 2010).

The exact function of viscosin in dispersal of *P. fluorescens* SBW25 biofilms was not elucidated in the present study. We speculate that biosurfactant-facilitated motility away from biofilm clusters should be preceded by modifications of cell surface hydrophobicity and the matrix structure to enable efficient dispersal. It has been suggested that lipopeptides affect cell surface hydrophobicity. However, a comparison of cell surface hydrophobicity of surface-grown SBW25 and SBW25Δ*viscA* by hydrophobic interaction chromatography did not show significant differences (data not shown). Hence, there is a need for future studies addressing how biosurfactant-supported motility interacts with mechanisms that change cell surface hydrophobicity and the biofilm matrix structure during dispersal. Interestingly, exogenous addition of viscosin interferes with microcolony formation and dissolves pre-existing biofilms of *P. aeruginosa* strains that do not produce lipopeptides (Raaijmakers *et al.*, 2010). This property is shared with other lipopeptides, e.g. putisolvin (Kuiper *et al.*, 2004), and it would also be of interest to unravel the mechanism behind this aspect of the impact of lipopeptide on biofilms.

In conclusion, the present study shows that the lipopeptide biosurfactant viscosin enhances dispersal of biofilms produced by *P. fluorescens* SBW25. Importantly, carbon starvation strongly induces *viscA* gene expression and subsequent biofilm dispersal. The mechanism behind viscosin-facilitated biofilm dispersal was not elucidated in the present study; however, a role for viscosin-facilitated motility is proposed. Future studies addressing the role of viscosin in biofilm dispersal will be of great interest, due to the industrial and clinical significance of developing novel biofilm-dispersing agents that can be used as disinfectants or surface-coating agents to prevent detrimental microbial surface colonization and biofilm development.
